# New Method for Automatic Body Length Measurement of the Collembolan, *Folsomia candida* Willem 1902 (Insecta: Collembola)

**DOI:** 10.1371/journal.pone.0098230

**Published:** 2014-06-05

**Authors:** Oxána Bánszegi, András Kosztolányi, Gábor Bakonyi, Borbála Szabó, Miklós Dombos

**Affiliations:** 1 Institute for Soil Sciences and Agricultural Chemistry, Centre for Agricultural Research, Hungarian Academy of Sciences, Budapest, Hungary; 2 MTA-DE “Lendület” Behavioural Ecology Research Group, Department of Evolutionary Zoology and Human Biology, University of Debrecen, Debrecen, Hungary; 3 Szent István University, Faculty of Agricultural and Environmental Science, Gödöllő, Hungary; 4 Szent István University, Faculty of Veterinary Science, Budapest, Hungary; Roehampton University, United Kingdom

## Abstract

The collembolan, *Folsomia candida,* is widely used in soil ecotoxicology. In recent years, growth rate of collembolans has become as frequently used endpoint as reproduction rate in ecotoxicological studies. However, measuring collembolan body sizes to estimate growth rate is a complicated and time-consuming task. Here we present a new image analysis method, which facilitates and accelerates the body length measurement of the collembolan *Folsomia candida*. The new software package, called CollScope, consists of three elements: 1) an imaging device; 2) photographing software; 3) an ImageJ macro for image processing, measurement and data analysis. We give a complete description of the operation of the software, the image analyzing process and describe its accuracy and reliability. The software with a detailed usage manual is attached as Supplementary Material. We report a case study to demonstrate that the automated measurement of collembolan body sizes is highly correlated with the traditional manual measurements (estimated measuring accuracy 0.05 mm). Furthermore, we performed a dose-response ecotoxicity test using cadmium-sulfate by using CollScope as well as classical methods for size measurement. Size data measured by CollScope or manually did not differ significantly. Furthermore the new software package decreased time consumption of the measurements to 42% when tested on 35 animals. Consequently, methodological investigations performed in this study should be regarded as a recommendation for any other routine dose-response study where body growth is an endpoint.

## Introduction

Over the past three decades the interest in ecotoxicological effects of new chemicals on soil organisms has been continuous. Several ecotoxicological tests have become widely used and are now commercially available. Soil ecotoxicology investigates the effects of chemicals on soil organisms in the environment [1]. There are three standardized ISO tests and one OECD guideline to test chemicals on the collembolan *Folsomia candida* Willem 1902 [2]–[5]. These tests aim at assessing toxicity expressed in terms of effects on selected endpoints like survival, reproduction or behavior based on dose-response relations [1]. On collembolans, the reproduction inhibition test is the most widely used [3], [6].

The protocol of the reproduction inhibition test characterizes the effects of a given toxic agent on the collembolan reproduction rate. This laboratory method is based on the direct visual identification of the number of offspring at the end of the test. The number of offspring may rise up to several thousand, therefore several automatized methods have been developed for this task [7]–[10].

Growth rate is a primary life-history characteristic, therefore it has also been suggested as another endpoint for toxicity tests. Reduced growth indicates the reduced fitness of the individual [11]–[13]. First, collembolans cannot reproduce until they gain a minimum weight [14]. Second, positive relationship was found between growth retardation and reduction in reproduction, probably due to lowered metabolic efficiency [15], [16]. The importance of growth-studies have been demonstrated repeatedly as they were able to detect reduction in growth caused by several different toxic agents [16], [17], including metals [15], [18]–[21]. Fountain & Hopkin [13] presented a test system by which the effects of xenobiotics on growth were noticeable in as young as 7-day old collembolans. Even though the growth test is slightly less sensitive to most of the chemicals compared to the reproduction test, it requires much fewer animals and is more rapid [13], [16], [22].

However, manual measuring of the collembolan’s length is time-consuming. For this process researchers initially used an ocular micrometer with magnification [23], [24]. Later, the most common method to measure a collembolan’s length was the methodology by Folker-Hansen *et al.* [17]. They introduced digital image processing equipment, which consisted of a stereo microscope mounted with a video camera. Pictures of the animals were displayed on the monitor while measurements were made manually using a digital pointing device. In the past few years digital cameras have become relatively cheap and free image analyzing software (e.g. ImageJ) can be used to measure the length of objects on the picture, so this method has become widely used. However, this method still requires a considerable amount of manual work in the laboratory. Collembola must be photographed first and then manually measured one by one on the images. According to the protocols, body length of adults is measured from the end of the posterior abdominal segment to the anterior margin of the head between the antennae, as described by Folker-Hansen *et al.* [17]. Since collembolans are soft bodied insects, they can be flattened or curved and some of their segments can be telescoped [25], the measurable length of the live animals may vary and as a consequence it is necessary to repeat both the taking of pictures and the measuring of individuals.

Our aim was to develop a digital imaging device and an image analysis software application linked to the procedure in order to measure the length of the living *F. candida* individuals automatically, which, at the same time, meets the accuracy demands of the body length measurement in ecotoxicological tests. We present here a new tool which can be used for constant monitoring of the collembolan growth throughout the exposure period in ecotoxicological experiments. We also report the accuracy of the method and its applicability in a standard ecotoxicological experiment.

## Materials and Methods

### Animals

*F. candida* belongs to Isotomidae family and is an eudaphic, non-pigmented, eyeless springtail with a parthenogenetic reproduction pattern.

*Folsomia candida* Willem 1902 (Collembola) was chosen as a model animal for our study because this species is widely used in soil ecotoxicology. We obtained a standard breed from the soil ecological laboratory of the Institute for Soil Sciences and Agricultural Chemistry, Centre for Agricultural Research, Hungarian Academy of Science. Collembolans were kept in 8 cm tall boxes with a base of 12×7 cm in constant darkness. 0.5 cm thick layer of plaster of Paris mixed with charcoal was added to the bottom of the boxes. The animals were fed 2 mg baker’s yeast once a week. The culturing boxes were opened once a week for airing and moistening with distilled water.

### Automatic Image Processing

Our software package consists of three elements: 1) the imaging device comprised of a digital camera with a lens and a light source; 2) software written in Delphi for automatizing the photographing; 3) an ImageJ macro for image processing, measurement and the data analysis. The whole method is called CollScope.

### 1) Imaging-CollScope Imaging Device

**Figure 1:**
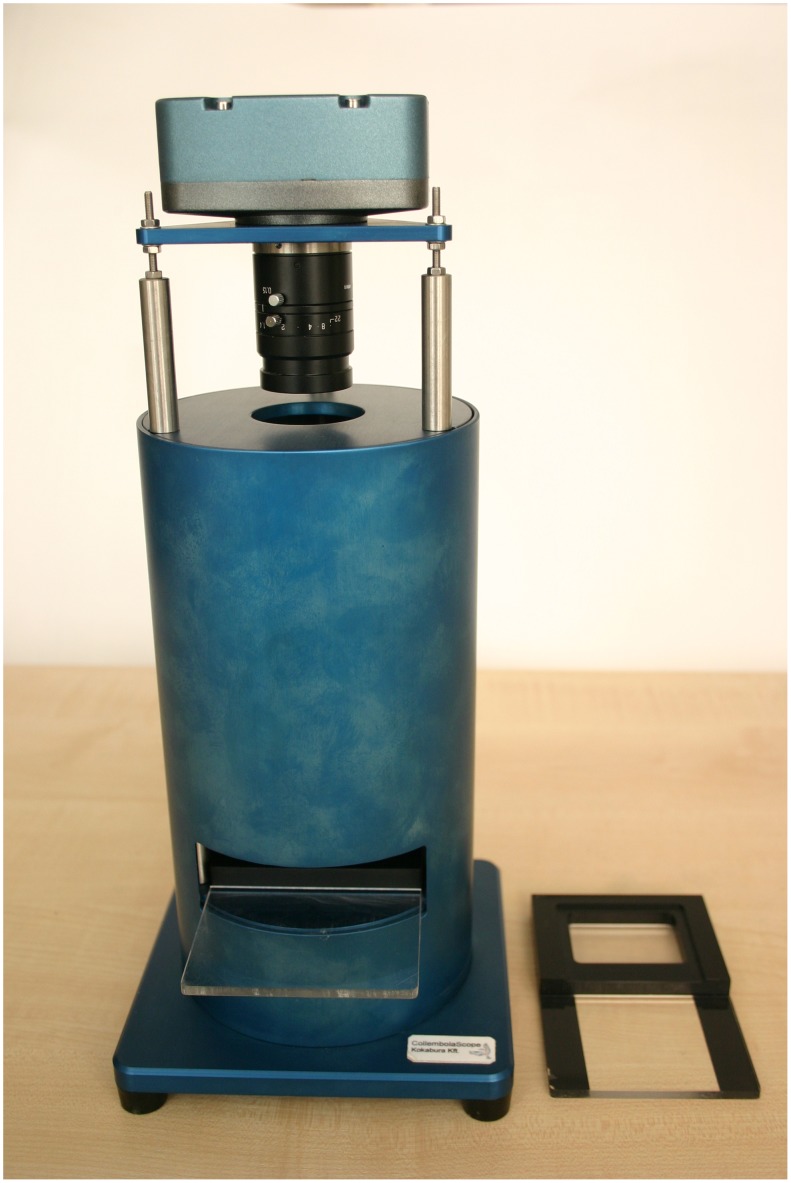
The CollScope imaging device. The device consisting of a 3.3-pixel Machine Vision CCTV lens, a sample holder with a view field of 48 mm×37 mm (bottom right) and uniform white backlight illumination.

The imaging device consists of a 3.3 MP color CCD camera (TCC-3.3ICE-N, ICX412AQ Sony CCD), a Tamron Mega-pixel Machine Vision CCTV lens (23FM25SP Focal length 25 mm, Aperture 1.4), a PMMA sample holder with a view field of 48 mm×37 mm, and uniform white backlight illumination. The resolution of the instrument can be characterized as one pixel corresponding to 24.6 micrometers * 24.6 micrometers. The microscope was enclosed in anodized aluminum housing to avoid the influence of external light (Figure 1). Sample holder position as well as lighting of the device needed to be completely stable during photographing; any changes resulted in false imaging and analysis.

### 2) Photographing the Animals–“CollScope Photographing” Software

The insects were removed from their boxes with an insect vacuum than were inserted into the PMMA plate, which was placed into the drawer of the microscope. Each plate contained one living individual of *F. candida*. All measurements were carried out in room temperature between 21–25°C. For this capturing process we developed a Delphi software, which simplified the image capturing and file storing processes. The software and its instructions can be found in Supplement S1. The software automatically captures ten consecutive images of each collembolan individual over a 30 second period in 3 second intervals and stores the images in JPEG format.

### 3) Analysis of Images–“CollScope Image Analysis” Software Application

**Figure 2:**
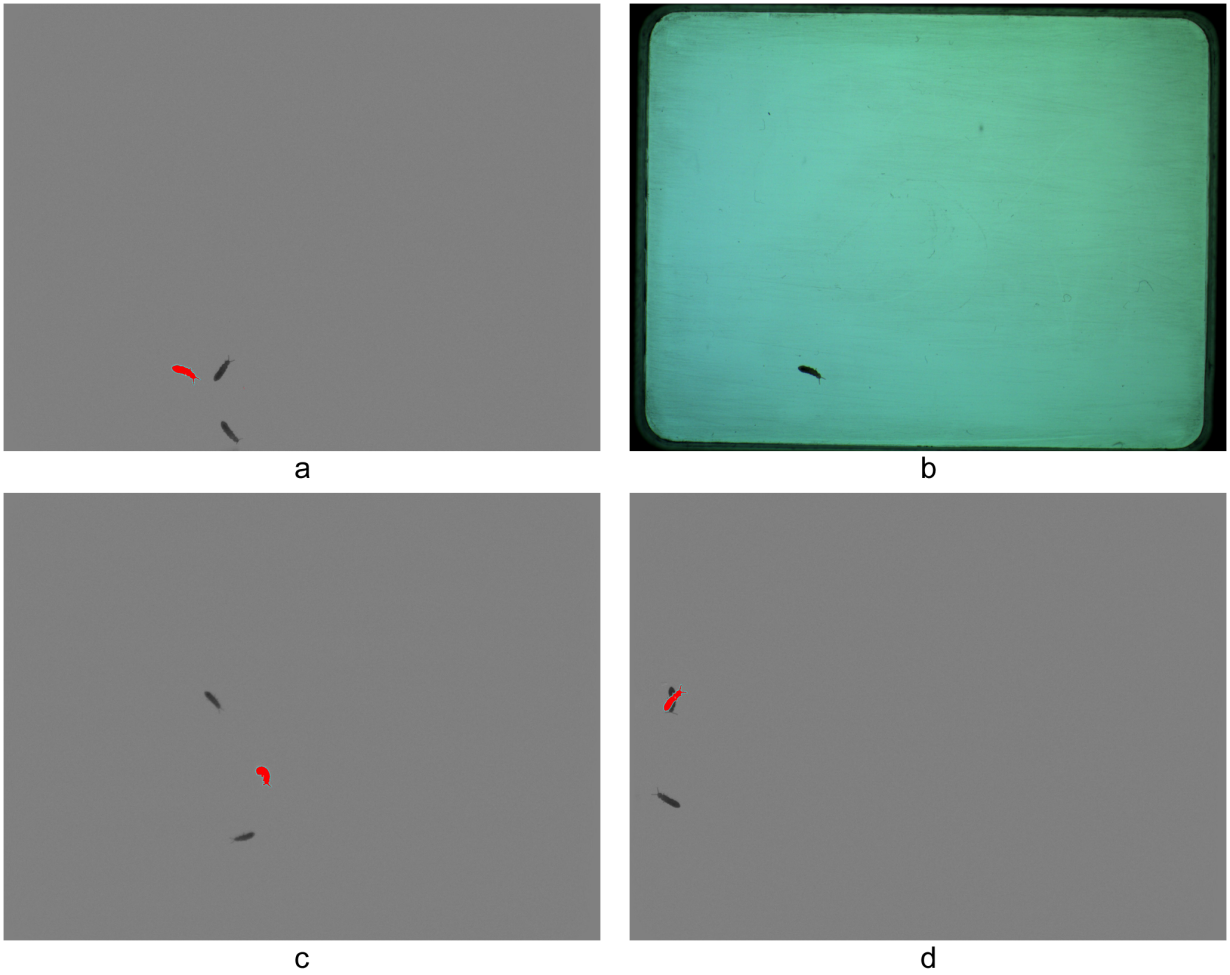
Secondary difference images produced by the CollScope image analysis software application. In the resulting pictures individuals appear in three representations: red patches correspond to the animal’s spot involved in further calculations, while dark grey patches show its previous and next positions (a). Original photo (b), Collembolan bending in a crescent on the image or split during the image processing (c and d).

In the image analysis process a major challenge was to distinguish the individuals from other, often overlapping particles like sand, excrement, vegetal particles etc. mixed in the field of view. In our method individuals are identified based on their movements during visualizing. In general, everything that changed position during the 30 seconds of image capture was treated as a live individual and everything else as background or noise that had to be excluded from the evaluation. During the analyses a pixel-extraction method was conducted resulting in patches corresponding to the moving animal. For this image analysis we wrote a plug-in for the open source image processing package ImageJ (version 1.48s; http://rsbweb.nih.gov/ij). Details of the plug-in and the plug-in can be found in Supplement S2. In short, the plug-in operates with differences of images. First, the difference between the current and previous image and the difference between the current and the next image is calculated, and then the two resulting difference images are used to create a final secondary difference image. Thus, out of the series of ten images, eight secondary difference images are created. On the secondary difference images, the displaced patches are in red, while the unchanged parts are in uniform gray. That is, if the animal moved, then it is a red patch on the image. Furthermore, the shots from the previous and next images are also showed in dark grey (Figure 2). These secondary difference images can then be thresholded and evaluated using the particle analysis tool of the image processing package ImageJ. Only particles within the 0.01 mm^2^–10 mm^2^ range are included in the image analysis. Although this plug-in calculates several parameters concerning the shapes of the patches, we used only the “Major” (M) to estimate the body length of the animals. This parameter corresponds to the major axis of the ellipse fitted to each patch of the picture. The result is exported into a simple text file. The file contains all data obtained from the eight secondary difference images. The table contains data calibrated in mm units; each row corresponding to one patch (see Supplement S2 for how to calibrate the system). Since there are eight secondary difference images for each animal, on average eight data rows can be obtained for each individual. However, there will be more patches and more M data if animals do not move sufficiently and therefore their patches overlap and their patches will be split, or there can be fewer, in case the animals do not move at all between images. If the animals do not move at all during the photographing, then no secondary difference images would be produced. However, since collembolans could be forced to move by air blowing, in our case this apparent drawback of the methodology did not cause serious difficulty.

### 4) Data Analysis–“CollScope Data Analysis” Software Application

#### Determining the accuracy and precision of the method

The raw M data stored in the text file required additional data processing, since they may contain errors occurring for two reasons: (a) inappropriate photographing or (b) biased length estimation from the patch.

- If the animal does not move sufficiently between two photographs, then only a part of its full size will appear on the secondary difference image. This means that the corresponding M parameter values obtained from these smaller spots will be lower, and the distribution of M values will be skewed. Furthermore, animals that are shot in an inappropriate position e.g. bending in a crescent or having a contracted shape will result also in a lower M value.
- As the plug-in fits an ellipse to the patches that takes into account the protruding appendages as well, we will always get a higher value for the larger diameter of this ellipse (i.e. the parameter M) compared to the body length measured between the head and abdomen manually.

First, errors caused by inappropriate photographing are corrected by data filtering. Since animals rarely stretched, the larger M parameter values are the more precise ones. Therefore the filtering process is based on the maximum value of the obtained M figures. We cannot use simply the maximum itself since collembolans are soft bodied and weakly sclerotized animals that do not have a stable body length. Therefore, we have to work with averaged data regardless whether an automatic or manual method is used. In this filtering process we determined a critical value below which we ignored the M data.

We analyzed images of 50 collembolans. Collembolans were randomly chosen from different rearing boxes; however, we tried to measure individuals of differing sizes. We analyzed the images using the plug-in, and then classified manually the original images whether the animal appeared in a regular or irregular position. We determined the critical value for the photographs of each individual as the lowest M value that corresponded to an animal in a regular position (i.e. in a position that would be regarded as appropriate in case of manual measurement). We divided this critical value by the highest M value of the individual. This ratio gives the critical ratio below which data are discarded and excluded from further analysis.

Second, the extent of the bias coming from the ellipse fitting was determined. In the sample of another 50 individuals, the length of the animals was manually measured on the first picture considered acceptable from the series of 10 pictures using the classic method (manual measuring of the animal’s body length on the screen from the end of the posterior abdominal segment to the anterior margin of the head between the antennae). The manual measurement was compared to the body length estimated by the M value of the corresponding secondary difference image received after the CollScope data analysis procedure, as described above. The relationship between the two variables was determined by linear regression, which gave a calibration equation. The estimated body length of an individual obtained by our method is the average of the filtered and calibrated M values. This part of the analysis also included in the ImageJ macro file.

Third, we tested the accuracy of our method using yet another 50 animals. In this test we measured the body length of animals both manually and automatically and compared the data coming from these two measurement procedures. For the classic manual measurements the first three pictures were used where the animal was in regular position and the measured body lengths were averaged. For the ScollScope method all 10 images were used.

### Application of the Device in an Ecotoxicological Test

A standard growth test on *F. candida* following Crouau and Moïa [16] was conducted, except that they used artificial soil, while in our experiment the animals were kept on plaster of Paris mixed with charcoal. Briefly, the effect of cadmium-sulphate (CdSO_4_) on *F. candida* growth was studied using synchronized animals. On day 0, 10–12-day old juvenile collembolans were introduced individually into plastic Petri dishes of 9 cm in diameter containing plaster of Paris mixed with charcoal on the bottom. Each dish was opened once a week for airing and moistening with distilled water. Collembolans were fed with baker’s yeast (about 2 mg per week). We used a control, and three nominal cadmium concentrations as follows: 0; 76.5; 153; 306 mg/kg dry soil CdSO_4_ (Sigma-Aldrich, purity ≥99%). Plaster of Paris was moistened with CdSO_4_ solution such that about 100% moisture content was achieved in the Petri dish. Each concentration had 15 replications resulting in 60 Petri dishes in all. Body length was chosen as an endpoint of the experiment. Measurements were taken on day 0, 7 and 14. Collembolans were measured manually as well as applying the new CollScope procedure at each sampling time.

We had repeated measurements from each individual, however, because of dropouts (6.7%, 6.7%, 13.3% and 53.3% of the individuals died during the experiment in the control, 76.5, 153 and 306 mg/kg cadmium concentration groups, respectively), the number of measurements varied between individuals. Therefore, we analyzed the data by linear mixed-models (LMMs) using the nlme package in R to account for the non-independence of repeated measurements. The effect of measurement method and cadmium treatment on growth over time was investigated in random intercept LMMs with individual identity as grouping factor in the models to control for the repeated measurements. In the models, the response variable was body length, whereas the fixed variables were the measurement method as factor (manual or CollScope), treatment as factor (control, 76.5, 153 and 306 mg/kg cadmium concentration) and day of measurement as factor (0, 7, 14 day). We also included treatment×day interaction term to investigate whether the growth over time was similar in the treatment groups. Models were fitted with maximum likelihood method and the effect of fixed variables was tested by likelihood ratio tests (LRTs). As there was a strong interaction between treatment and day of measurement (see results), we further analyzed the effect of treatment for each measurement day separately by one-way ANOVAs. Because the measurements of Manual and CollScope method was not different (see results), in the ANOVAs the measurements of CollScope method were used.

## Results

In the first study, from the 50 collembolans 408 M data were produced by Collscope Image analysis instead of the expected 400. On average, we obtained 8.16±1.54 (mean ± SD) M data for an individual. 285 out of the 408 pictures were evaluated as appropriate shoots, that is, the animals’ movements were sufficient and animals were pictured in a straight body position on these images. On average, for each animal 5.7±1.36 (mean ± SD) M data were classified as appropriate. The mean critical ratio obtained was 0.939±0.027 (mean ± SD from the 50 animals). That is M data less than 93.9% of the highest M value obtained from image analysis of the eight secondary difference images has to be omitted from the determination of body length. The 93.9% threshold value was built into the CollScope data analysis.

**Figure 3:**
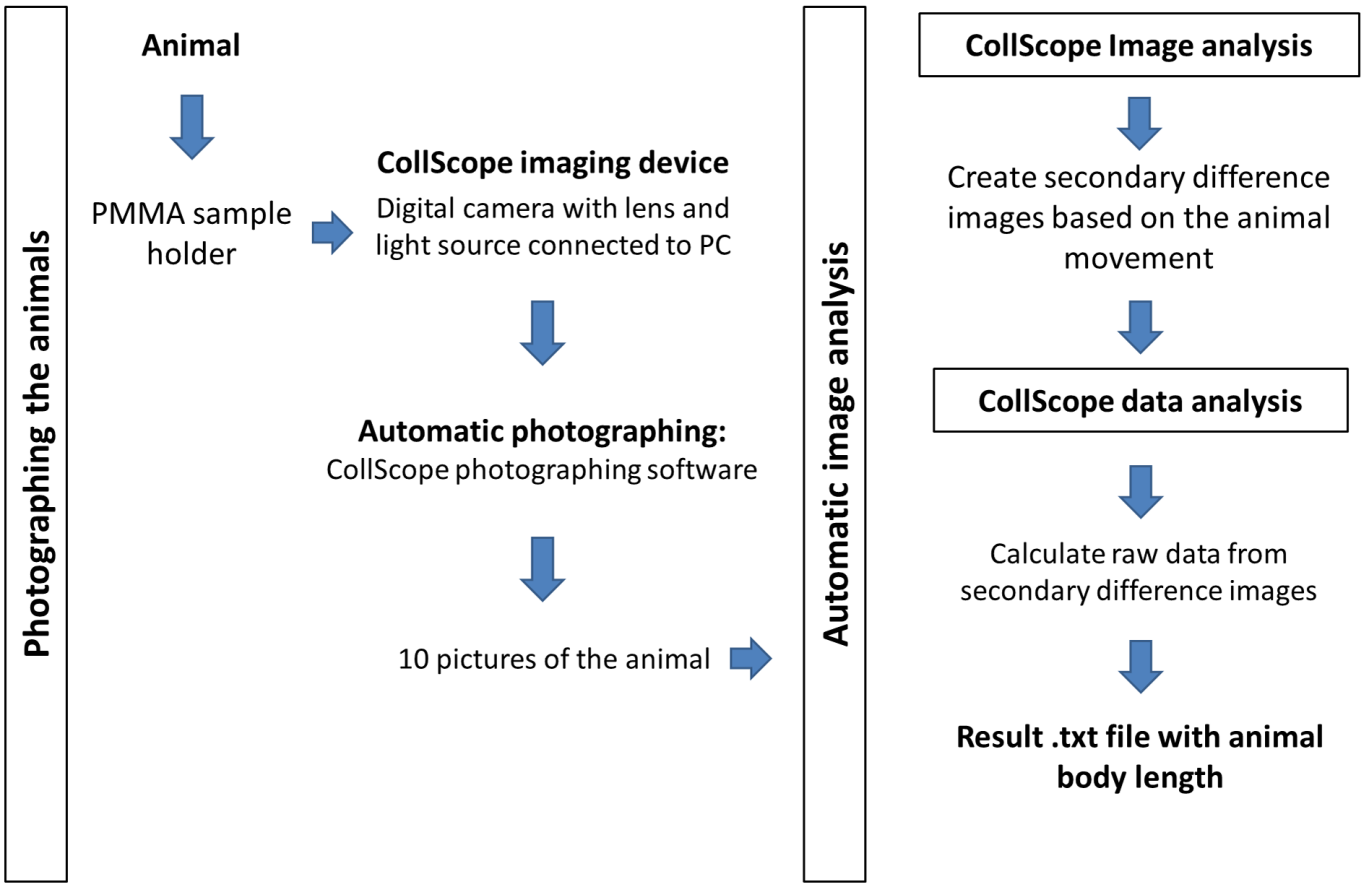
Flow chart of the CollScope method.

**Figure 4:**
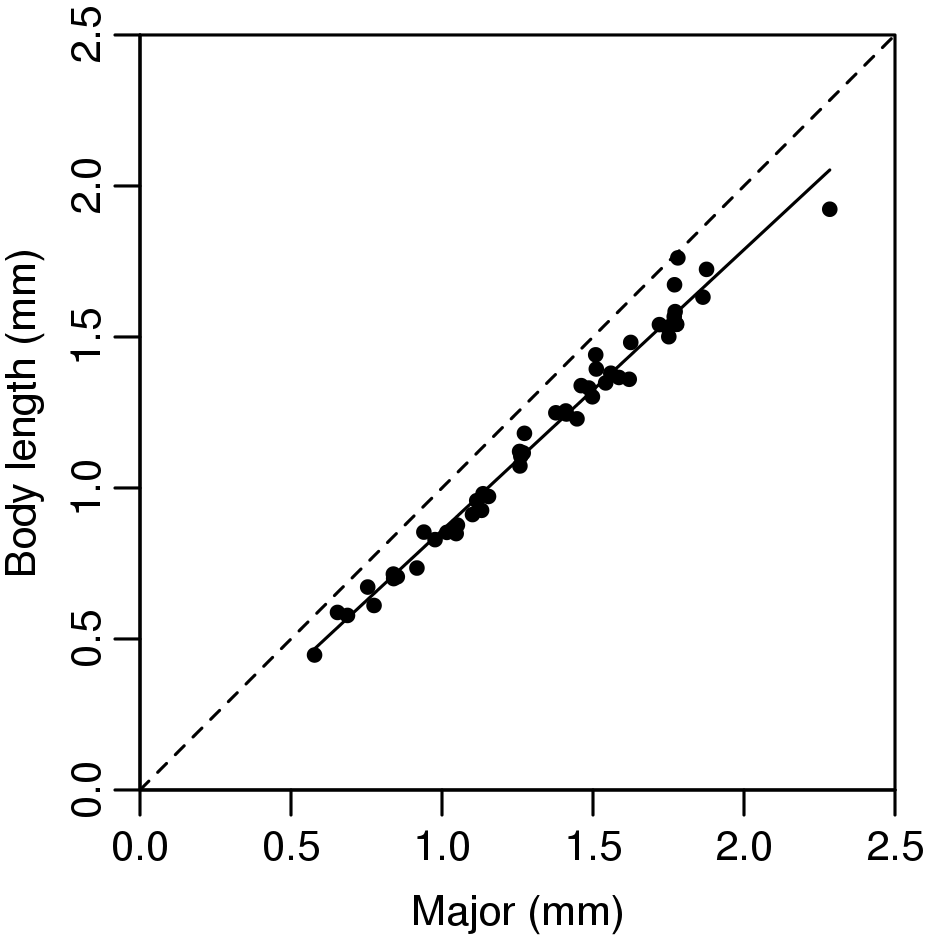
Relationship between the M values obtained from the image analysis and body length measured manually. The broken line corresponds to y = x. The continuous line shows the prediction used in the calculations (n = 50 individuals).

The regression of the manually measured body lengths of the second set of 50 individuals to the Major value gave the calibration equation: body length = 0.930×Major −0.070 (R^2^ = 0.98). The negative intercept indicates that the ellipse fitting slightly overestimated the true body length, whereas the slope smaller than one indicates that the bias is size dependent (larger individuals were overestimated more, Figure 3). This calibration equation was built into the ImageJ macro file. The result of the third study shows that the matching between the classic manual measurement and our newly developed method was significantly correlated (Pearson correlation: r = 0.99; n = 50; p<0.001) (Figure 4). In the ecotoxicological test no collembolan mortality was observed in the control group on day 7 and it was 6.7% at the end of the experiment. Consequently, experimental conditions can be regarded as appropriate during the experimental period.

**Figure 5:**
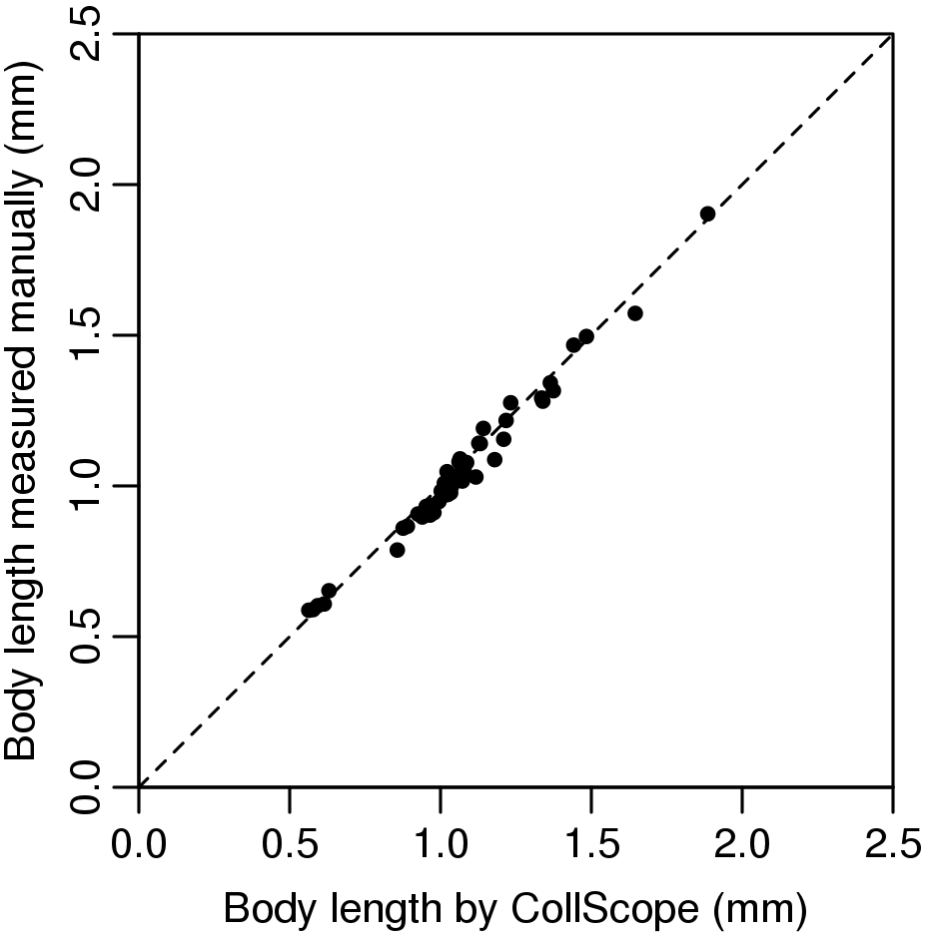
Accuracy of the CollScope method. Relationship between the body length estimated by CollScope and measured manually (n = 50 individuals). The broken line corresponds to y = x.

**Figure 6:**
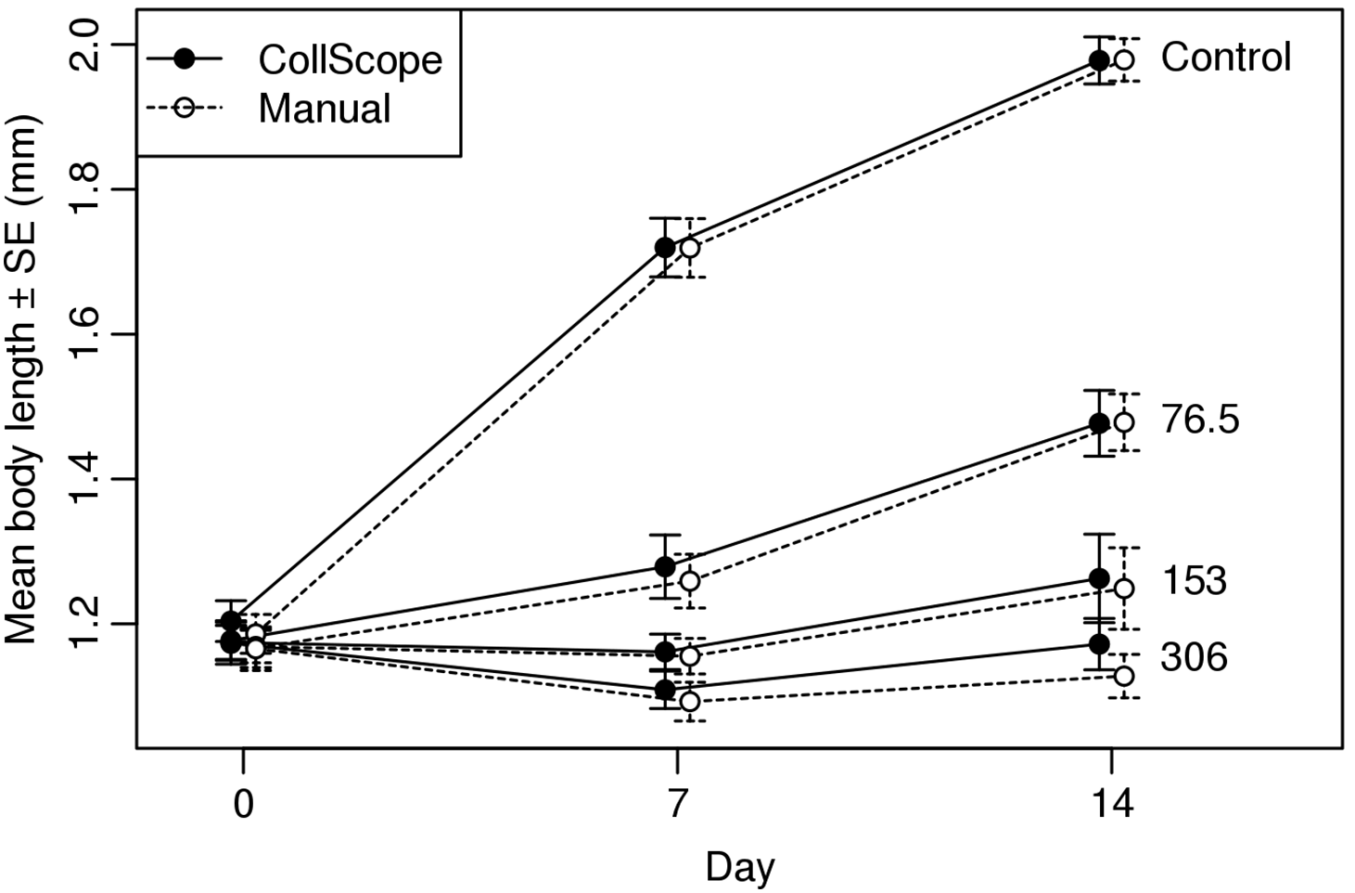
Body length of *F. candida* (mean± SE) exposed to different CdSO_4_ concentrations at sampling days 0, 7 and 14. Open circles correspond to manual measurements, closed circles to CollScope measurements.

Body length measurements did not differ between the two methods over the experiment (Figure 5, LRT, χ2 = 1.36, df = 1, p = 0.244). There was a very strong interaction between treatment and day of measurement (Figure 5, LRT, χ2 = 396.84, df = 6, p<0.001): there was no difference between treatment groups at the beginning of the experiment (day 0: ANOVA, F_3,56_ = 0.29, p = 0.833), whereas body lengths were significantly different on days 7 and 14 (ANOVAs, day 7:, F_3,47_ = 58.27, p<0.001; day 14: ANOVA, F_3,44_ = 59.95, p<0.001) (Figure 6).

## Discussion

In the first study we developed a data filtering process and a calibration equation to estimate the body length of *F. candida*. Optimally, 400 M data were to be obtained for the 50 collembolans (8×50), however, 408 M data were produced. These extra 8 data must be because either some individuals did not move sufficiently during the photographing therefore their secondary images showed two or more patches (increase in the number of M data), or the animals did not move at all between three images and their secondary images contained no patches (decrease in the number of M data). Since on average we obtained out of the 8 M data generated for one collembolan 5.7 M data appropriate for analysis, we can conclude that the 3-second time interval for photographing was usually sufficient for the animals to completely change their position, which is essential for proper image analysis.

In the third study, we showed that the body length values measured with the new image analysis method and those of the classical manual method were highly correlated. The results show that our newly developed digital imaging device with the image analysis software can be used for the accurate measurement of living collembolan body length.

Measuring the growth of individuals as an endpoint in ecotoxicological studies is increasingly widespread. Improvement in the measurement of the body length of the animals both in accuracy and speed has long been needed [16]. As presented here, using the CollScope software package the same degree of accuracy can be reached as with the classic manual measurement. However, it is less time-consuming, since the photographing method, selection of appropriate images and the measurement itself are each automatized.

By using the new CollScope software package we were able to reduce time period of the measurement to 42% (n = 35 individuals). In our test the minimum period of time necessary for the apparatus to take photos was 30 seconds, which can be modified according to the number of pictures set in the CollScope Shooting Software. If the animals are more or less mobile the time intervals between two pictures can be adjusted, as well. In addition to that the phase of putting the animals from the culture pot to the plate of the CollScope digital microscope and vice versa remained as the only time consuming and human involved part that lead to a method less sensitive to human error.

Although the growth of *Folsomia candida* has a lower sensitivity in toxicity tests than reproduction [20], [21], it has become a complementary variable [13], [16]. In a standard ecotoxicological study the endpoints are mortality and/or reproduction. A major disadvantage of reproduction tests is that the effects of the toxic agent will only be seen at the end of the exposure period, which is usually 28 days in the case of *F. candida* [3]. In contrast, some metal effects on body growth can be observed as early as 7 days after the beginning of the experiment [13]. Our results confirm the previous findings, that cadmium-sulphate reduces growth by day 7. And this result is consistent with the results found by Crommentuijn et al. [26].

Reproduction an important biomarker for the level of toxicity of a compound, however, in some cases, growth may be more sensitive and, therefore, more useful to provide an early warning system [17]. Van Straalen *et al.* [27] found in female *Orchesella cincta* that cadmium reduces the growth of individuals without affecting their reproduction. Toxic agents can influence an animal’s fitness in several ways, some of the effects are more quickly measurable others are not. Hence, it is suggested to always consider a number of endpoint variables. Additionally, the decrease of the density in a population can occur not just via the lack of the production of juveniles, but via the impaired growth of the juveniles, which delays egg production [14], [18], [23], [24].

We also demonstrated the application of the method in a standard ecotoxicology test that confirmed that the CollScope software package can be used in these types of experiments. Body length measurement has been performed on the same individual with both methods and the results showed no significant differences between the methods. This new method can be used for constant monitoring of Collembolan growth throughout the exposure period in ecotoxicological experiments.

Our methodology for body length measurements of *F. candida* by automatized processes provides a less labor-intensive way to carry out ecotoxicity tests and further ecological investigations. This methodology can be set up by end-users using a digital camera mounted on a stereo microscope; or by our professionals. Software can be downloaded for free. In this way, these ecotoxicological and ecological tests become time-effective.

## Supporting Information

Supplement S1 **“CollScope Photographing” software and its documentation.**
(RAR)

Supplement S2 **CollScope image analysis macro and its documentation and calibration guide.**
(RAR)
